# Secure lightweight cryptosystem for IoT and pervasive computing

**DOI:** 10.1038/s41598-022-20373-7

**Published:** 2022-11-16

**Authors:** Mohammed Abutaha, Basil Atawneh, Layla Hammouri, Georges Kaddoum

**Affiliations:** 1 Palestine Polytechnic University, College of Information Technology and Engineering, Hebron, Palestine; 2 Département de génie électrique, École de technologie supérieure, 1100, rue Notre-Dame Ouest, Montreal, Canada

**Keywords:** Energy science and technology, Engineering, Physics

## Abstract

Large volumes of sensitive data are being transferred among devices as the Internet of Things (IoT) grows in popularity. As a result, security measures must be implemented to ensure that unauthorized parties do not obtain access to the data. It is well acknowledged that IoT devices have restricted resources, such as limited battery life, memory, and hence reaction time. Classical encryption approaches and methods become inefficient for IoT devices due to memory limits. Large volumes of sensitive data are being transferred between devices as the Internet of Things (IoT) grows in popularity. This involves the implementation of security safeguards to ensure that unauthorized parties do not obtain access to the data. IoT devices are notorious for having limited resources, such as battery life, memory, and hence response time. Classical encryption approaches and methods become inefficient for IoT devices due to memory limits. As a result, a Lightweight cryptosystem that fits the needs of Lightweight devices and ubiquitous computing systems has emerged. The goal of this study is to present a Lightweight cryptosystem (LWC) that may be used as a plugin to secure data transfers in IoT devices and pervasive computing. To that goal, the researchers employ several simple measuring techniques. The suggested system was then implemented on a field-programmable gate array (FPGA) board using the Verilog programming language to demonstrate its appropriateness for actual security applications. FPGA is also utilized in hardware applications to assess the system’s resource usage and performance. Finally, a comparison of the proposed system with previous lightweight cryptography systems is performed to reinforce the major goal of this work, which is to present a new lightweight cryptosystem.

## Introduction

With the incredible growth of technology over the previous century, computers, machines, and gadgets have been able to perform some functions without the need for human participation. IoT^1^ refers to these devices linked as a network of equipment, gadgets, and computers that may communicate with one another. IoT also enables data transport and sharing via a network without the need for direct human engagement. IoT may be utilized in a variety of sectors in our daily lives, including healthcare and industry. The relevance of IoT is growing fast, and it is managing many aspects of our lives; thus, it is critical to preserve and secure the information and data utilized in IoT^2,3^.

To preserve and protect sensitive material, one must encrypt it. Encrypting data increases security by prohibiting unauthorized parties from accessing or modifying it (integrity constraints). Encryption is a method of hiding original data by using a specific key; this process turns the original data into another shape that hides the original data’s essence. In this process, the original data is referred to as plaintext, while the encrypted data is referred to as ciphertext. The encryption process should be reversible, allowing the plaintext to be recovered from the ciphertext with the help of a key. If the same key is used for encryption and decryption, the encryption is symmetric. If, on the other hand, the key used in decryption is different from the key used in encryption, the encryption is asymmetric. In asymmetric encryption, one key is private while the other is made public. The public key of the receiver is used for encryption, whereas the private key of the receiver is utilized for decryption^4^.

Internet of Things (IoT) devices include wearable devices, monitors, sensors, and any other computer-capable item^5^. These gadgets often feature a small memory capacity, a short battery life, and a quick response time . Because of the resource restrictions of IoT devices, they necessitate a specialized encryption algorithm that saves energy usage, accounts for limited accessible memory, and maintains a quicker response time^6^. Lightweight cryptosystems are the name given to these specific cryptosystems.

The suggested system extends the previous stream cipher chaos-based encryption method created by Abutaha et al.^7^. This chaos-based encryption technique is extended to create a LWC. This chaos-based encryption technique is extended to create a LWC. This chaos-based encryption method is expanded to produce a LWC. This study’s key contribution is the development of a LWC system that may be employed in embedded systems, IoT devices, or any other device with limited resources.

In our new work we present an effective LWC with software and hardware implementation on a field-programmable gate array (FPGA) board using the Verilog programming language to show its suitability for practical security applications. In addition, FPGA is used in hardware applications to evaluate the system’s resource utilization and performance. In addition, in our new work, we apply the RAPL and POWERTOP to calculate power usage. In addition, we included memory consumption, resource performance, and utilization in our comprehensive research.

The “Background and literature review” section explains the key principles and reviews past work on this topic. Measurement instruments for power and memory are mentioned in the “Design and methodology” section. The “Software and hardware implementation” section describes the system’s software and hardware implementation. Under the meanwhile, the findings are displayed in the “Results” section. Finally, the “Conclusion and future work” section analyzes the findings and recommendations for further work.

## Background and literature review

### Background

#### Encryption

**Figure 1:**
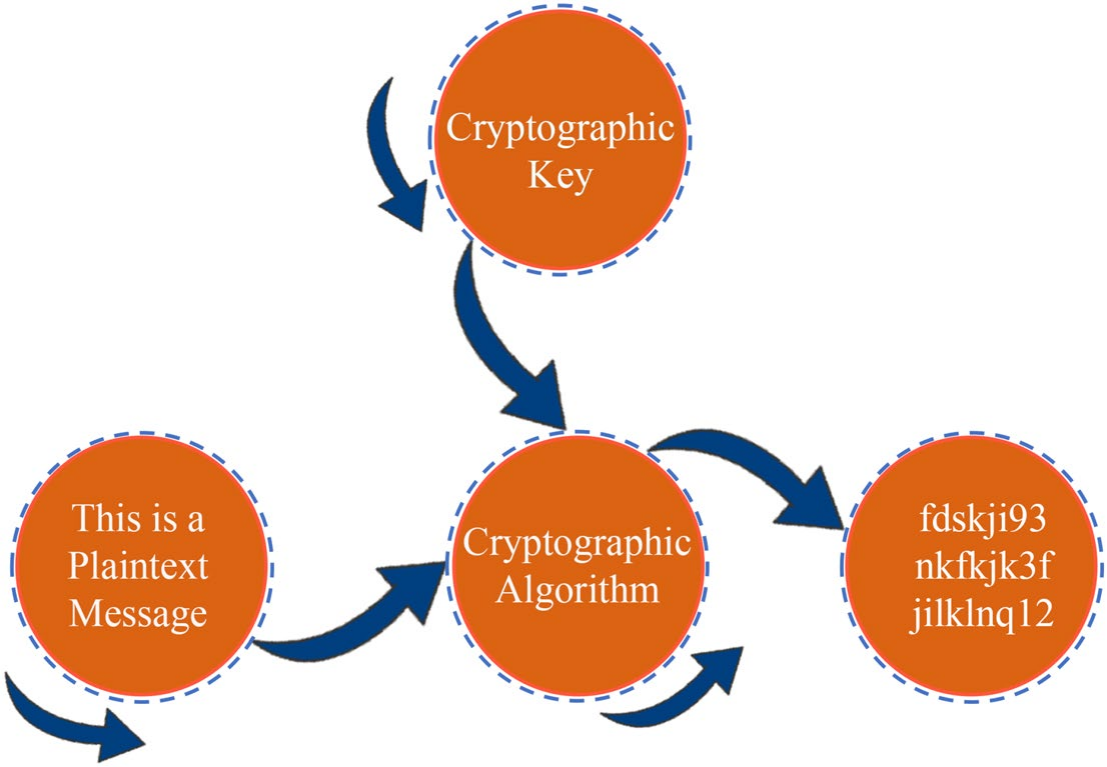
Encryption and decryption process using a key.

Encryption, as seen in Fig. 1, is a method used to conceal the original data (plaintext) by transforming it into secret data (ciphertext) using a secret key. To safeguard the original data (plaintext), the ciphertext does not reveal anything about it. As a result, only the sender and recipient have access to the original data. Cryptography is the study of encryption and decryption^8^. To maintain data security, most apps, computers, and devices (through IoT) employ encryption in their communication. The data is encrypted using a secret key to create the ciphertext, which is then sent to the recipient. The key is known to the recipient, who uses it to decrypt the ciphertext and obtain the original data. If the key used to encrypt and decode the data is the same, the encryption is symmetric, however in asymmetric encryption, we can encrypt the entire data set at once, which is known as stream cipher. Otherwise, we can partition the data into blocks, encrypt each one, and then transmit them as blocks to be decrypted. Block cipher is the process of dividing data into blocks to be encrypted.

#### Block cipher

As previously stated, block cipher encryption is a symmetric encryption that encrypts n-bits of data at a time to generate n-bits of ciphertext; each n-bit is referred to as a block. If the data size is greater than n bits, it can be partitioned into blocks that generate a block of ciphertext for each block of data. After partitioning the entire data into blocks based on block size, if some remaining bits do not match the block size, padding techniques are used to finish the block so that the remaining bits may be encrypted as a block. 64 bit, 128 bit, and 256 bit are the most frequent block sizes. There are numerous block cipher algorithms available, including Digital Encryption Standard (DES), Triple DES, Advanced Encryption Standard (AES), and others^9^. Furthermore, depending on the mode characteristics, several modes of operation are utilized in a block cipher for different purposes.

#### Stream cipher

Stream cipher is another sort of Symmetric encryption in which we encrypt 1 bit or byte of plaintext at a time. There are several stream cipher algorithms, such as RC4^10^, salsa20^11^, and Rabbit^12^.

Stream cipher inventors worked hard to develop the desired cipher, known as the One-Time pad, in which the key is equal to or greater than the plaintext length. A mathematical equation with two unknown values is used in one-time pad encryption. These are the key and plaintext values. Attempting to solve the equation to obtain the key or plaintext with only the ciphertext is theoretically impossible; this is why one-time pad encryption systems guarantee unbreakable encryption in use. Without the right key, it is difficult to decipher the encrypted data using one-time pad encryption^13^.

The key distribution issue with the One-Time Pad (OTP) is the fundamental issue. To make the OTP feasible, we must exchange a random (not pseudo-random) key of the message length. One technique (and maybe the most promising) to tackle the problem of sharing a large enough, random key to use with the OTP is using Quantum Key Distribution (QKD)^14,15^.

#### Pseudo-random number generator (PRNG)

The stream cipher generates a series of random integers using mathematical algorithms. This sequence is the produced key value. The numbers in the series almost have the qualities of random numbers, making them difficult to predict. The pseudorandom generator must be unexpected, which implies there should be no effective technique that can predict the next key from the preceding one with a probability greater than 0.5^16^.

#### Internet of things (IoT)

The machine-to-machine (M2M) concept was created in the late 1970s to explain wired or wireless communication between devices. This notion was then used to a variety of applications, including data collection via sensors. With the growth of the internet in the 2000s, the M2M idea brought the “IoT,” which stands for “Internet of Things.” In this environment, “Things,” which may be apps, machines, sensors, cars, and so on, can communicate with one another without the need for user intervention, and they can freely transmit data^17^. It’s a given that “Things” use a computer to communicate with one another. This idea gave rise to the phrase “ubiquitous computing”.

Ubiquitous computing indicates that computers are everywhere, and they may be any device, in any place, and in any format at any time. Pervasive computing, wearable computing, and tangible computers are examples of ubiquitous computing.

#### Lightweight

All of the Ubiquitous computing shapes discussed above have one thing in common: they have a limited battery life, a small memory space, and a quick response time. This is due to the fact that they are not stand-alone computers; they are part of something larger, such as a machine or other hardware.

The phrase ‘Lightweight software’ was created by combining speed limitations, memory use constraints, and energy consumption constraints.

Sensors, for example, are classified as IoT devices and are known to be resource-constrained. As a result, the lightweight notion is critical to their work. Since a result, softwares, algorithms, programs, protocols, and applications should be developed on lightweight standards in order to operate efficiently when implemented in IoT devices, as they get greater performance when they are lightweight based.

#### Lightweight cryptography (LWC)

With the expansion of IoT, there is a lot of sensitive information being sent between machines and devices, and we need to encrypt this data to ensure its safety and confidentiality. As previously stated, encryption is the process of modifying (encrypting) data so that it cannot be accessed by unauthorized people. Unfortunately, most existing encryption algorithms and strategies were designed to secure connections between desktop computers and devices with substantial resources, and hence do not operate in resource-constrained devices, such as IoT devices^18^. Classical encryption techniques can be overly sluggish, reducing reaction time, and they can also waste a lot of power. As a result, lightweight encryption approaches are required.

Lightweight encryption should meet the primary purpose of encryption, which is to secure data between sender and receiver, while also ensuring that this process is done at a lower cost and power consumption than existing non-lightweight encryption methods^19^.

#### Verilog

Verilog is a Hardware Description Language (HDL) that gives an electrical description of system behavior and structure, as well as circuit designs. The language’s behavioral degree of abstraction serves the early conceptual phases of design, while structural abstractions support the later implementation stages^20^. Verilog is used for simulation, timing analysis, test analysis, and circuit synthesis^21^.

#### FPGA

Field Programmable Gate Arrays (FPGAs) are integrated circuits that can be modified after they have been developed by a manufacturer. In most cases, a hardware description language (HDL) such as Verilog is used to define the FPGA setup. FPGAs may be reconfigured after they have been manufactured to fulfill specific application or feature requirements. Although one-time programmable FPGAs are accessible, the large proportion of FPGAs are founded on Static Random Access Memory (SRAM) and may be reprogrammed as the modifications^22^.

### Literature review

This research focus on three pseudo-chaotic number generators (PCNGs) that employ basic chaotic maps. Additionally, it explored three chaotic stream ciphers depending on the evaluated PCNGs. The scientists also analyzed the energy, power, and processing time of the chaotic systems in investigation using an Real Time Operating System (RTOS) named Xenomai. The study stressed the need of secure data transmission in IoT devices in order to ensure data security. Implementing traditional encryption algorithms on low-resource devices would need massive quantities of storage, power, and process time, which is inconsistent with embedded capabilities. Lightweight cryptosystems are required to deal with the scarce funds of IoT devices. We may deduce that the cryptosystems under examination are lightweight cryptosystems by comparing the findings to other cryptosystems and examining the energy and power consumption parameters. The investigation also shown that the generated stream ciphers are suitable for use in IoT devices as well as real-time cryptosystems based on an RTOS called Xenomai, according to the environmental resources.

#### Lightweight cryptography

The Cryptography Research and Evaluation Committees (CRYPTREC)^23^ clarify what Lightweight cryptography is and why it is required. The Internet of Things (IoT) is becoming more popular, and there are a growing number of low-resource devices connecting to the internet. When using these limited-resource devices, we must ensure security and privacy.

Traditional encryption systems have been developed into Lightweight cryptography systems to care for cost, energy, and latency.

To account for cost, energy, and delay, conventional encryption methods have indeed been transformed into lightweight cryptography systems. A lightweight cryptosystem is one that may be implemented in either hardware or software. In hardware implementations, circuit size is important since it has a direct influence on cost, power consumption to preserve energy, and other things. The amount of time necessary to perform encryption or decryption procedures is referred to as latency. It should be mentioned that real-time operating systems and computers demand low latency. Because embedded device CPUs have limited RAM and ROM, memory storage is important for program development^23^. Several block cipher encryption and stream cipher algorithms are evaluated and compared with CRYPTREC study to determine the benefits and drawbacks of each technique. This article^23^ presents many cryptosystems, including ChaCha20 (2008), Enocoro (2008), Grain v1 (2005), and Trivium(2005), and afterwards discusses their characteristics, security analysis, decision alternatives, Standardization, industrial adoption, and etc.^23^.

#### New lightweight stream cipher based on chaos

Lina Ding and his colleagues^24^ used 80-bit encryption techniques to create a hardware-oriented lightweight stream cipher approach based on chaos (Logic stream cipher). A logistic chaotic map, two Nonlinear Feedback Shift Registers (NFSR), a filter function, and a multiplexer unit are all part of the chaotic technique. The Logistic chaotic sequence is mixed with multiplexers and NFSRs after digitalization. In this research, the Logic stream cipher was tested against a number of lightweight cryptographic algorithms, and it was discovered that it performed well, with a throughput of 78.98 Kbps at 100 kHz and good complexity.

#### Lightweight chaos-based stream cipher hardware implementation

The study describes a hardware implementation of a chaos-based stream cipher. The hardware implementation is proposed in^24^ for the Zynq7000 platform. This implementation is focused with throughput performance and logical resource utilization. The system is compared to earlier stream cipher hardware implementations.

The system’s basic foundation is a generator composed of two cells that rely on two separate Chaotic maps known as the Skew Tent map and the PieceWise Linear Chaotic (PWLC) map. To acquire the stream key, the results of the two maps are XORed.

This system’s findings are compared to those of other stream ciphers in terms of performance, resource utilization, and speed. The Xilinx Vivado tool is utilized for this performance comparison. A bit depth (N) of 32 bits and a delay (D) of 3 were employed in the comparison. This system solution has a throughput of 565 Mbps and can run at 18.5 MHz.

#### Chaotic encryption algorithm design and implementation for real-time embedded systems

In their article, Amit Pande and Joseph Zambreno^25^ presented a new chaotic encryption technique for real-time embedded devices.

The system uses the Modified Logistic Map (MLM) as a pseudo-random number generator, which was created by changing the Logistic Map to increase uncertainty. They analyzed various current chaotic ciphers to ensure that the new modified system can withstand known assaults before proposing this new chaos-based stream cipher system. The system’s hardware implementation was then tested on the Xilinx Virtex6 FPGA. The hardware implementation, which used 16 hardware multipliers, produced a synthesis clock frequency of 93 MHz and a throughput of 1.5 Gbps. The results show that the system is capable of meeting real-time needs.

## Design and methodology

This section provides an overview of different measuring techniques for power and memory consumption that are suitable for LWC. In addition, we talk about the improved system. Finally, the suggested system’s implementation on an FPGA-board is detailed.

### Encryption system based on chaos

**Figure 2:**
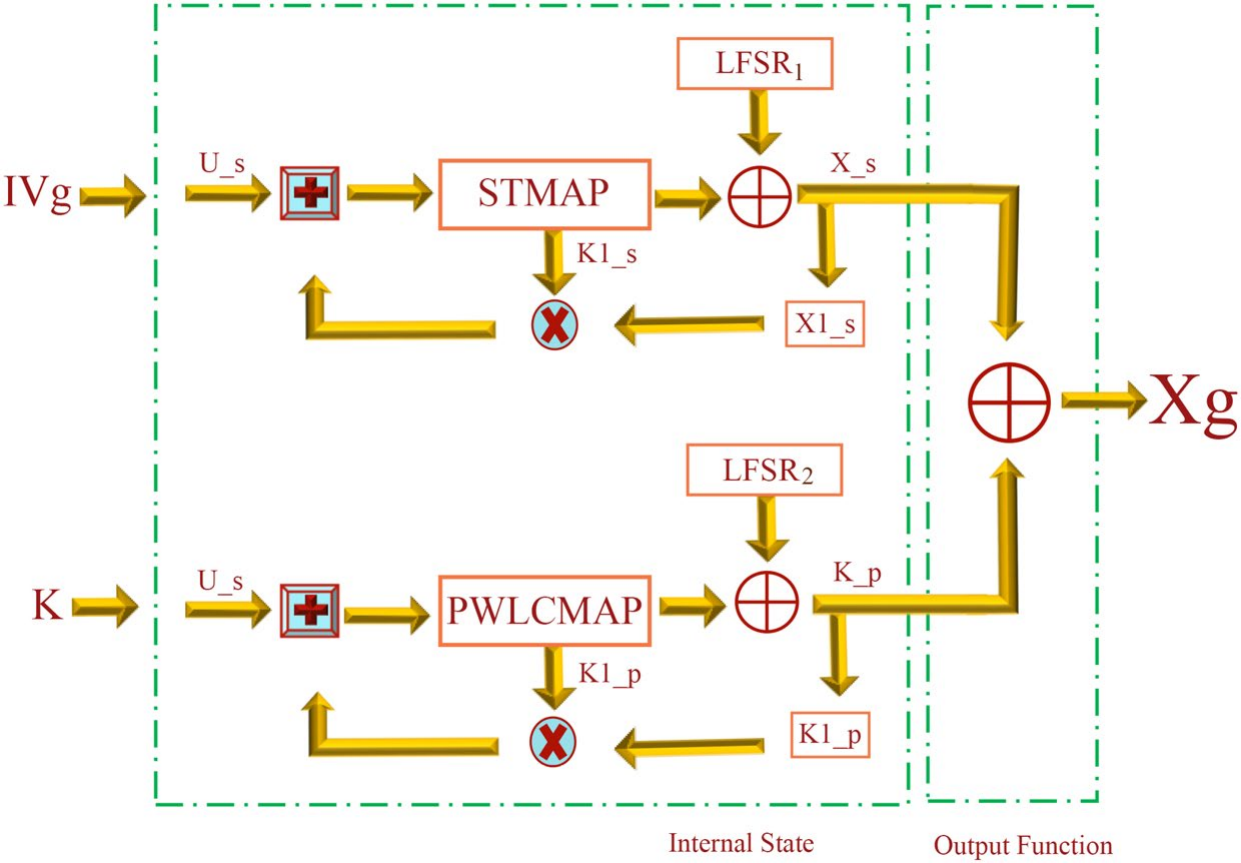
The detailed structure of the key stream generator.

**Figure 3:**
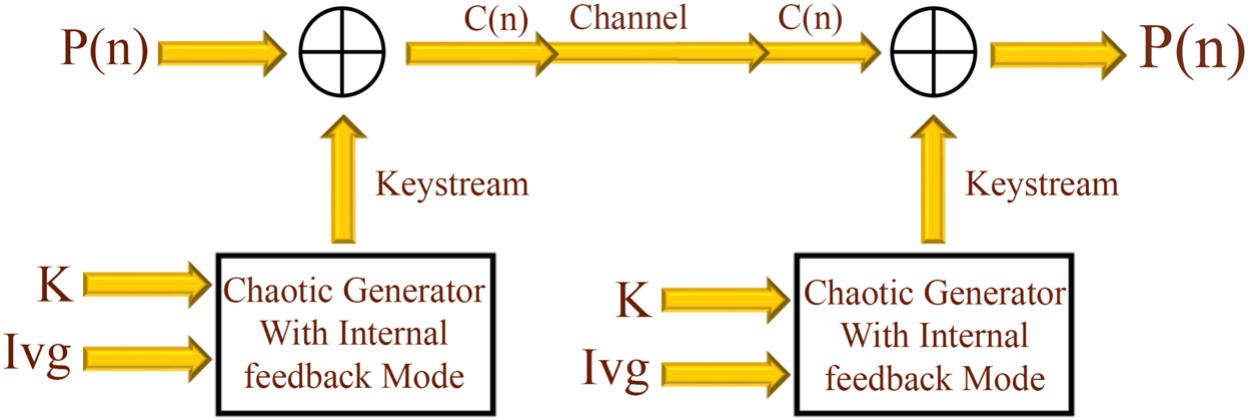
General Stream cipher structure with internal feedback mode.

The ciphering method is based on chaos, which is a state of dynamical systems characterized by seemingly random states of disorder and irregularity that are frequently restricted by deterministic rules that are very sensitive to initial conditions. In order to acquire the ciphering data, the key-stream generator will construct the proper key streams for each syntactic element. Our previous work lends support to the key stream generation^7^. The internal state, which holds the majority of the cryptographic complexity of the system, is composed of two third-order recursive filters. The first recursive cell employs a discrete Skew tent map, while the second employs a discrete piecewise linear chaotic map. These chaotic maps are created using non-linear filtering. With each key-stream generating call, a new beginning vector IV value is generated. This quantity creates a unique key stream sequence for each generation call. In^7^, designers elaborate on the key stream generator’s cryptographic security analysis and thorough construction. Figure 2 displays the chaotic map-based key stream generation. This proposed method encrypts the frame’s information bytes using a chaos-based stream cipher. The concept of this encrypting system is based on rapid chaotic generation, which employs two chaotic non-linear recursive filters, a disruption mechanism, and a chaos-based multiplexer. It uses a stream cipher to encrypt data. In practice, a chaotic generatorbased synchronous cryptography approach was used. Both the emitter and receiver require the shared secrete key in order to use the chaos-based generator and generate the key-streams required by the encryption and decryption procedures^26^. Figure 3 depicts the construction of this encryption scheme. The formulae for the Discrete Skew Tent and Discrete PWLCM maps are provided by^7^: Discrete Skew Tent Map:

1 $$\begin{aligned} X{_s}[n]={\left\{ \begin{array}{ll}\left\lceil 2^N \times \frac{X{_s}[n-1]}{P1} \right\rceil &{} \text {if } 0<X{_s}[n-1]<P1 \\ 2^N - 1 &{} \text {if } X{_s}[n-1]=P1 \\ \left\lceil 2^N \times \frac{2^N - X{_s}[n-1]}{2^N - P1} \right\rceil \ &{} \text {if } P1<X{_s}[n-1]<2^N \end{array}\right. }. \end{aligned}$$

Discrete PWLCM map:

2 $$\begin{aligned} X{_p}[n]={\left\{ \begin{array}{ll}\left\lceil 2^N \times \frac{X{_p}[n-1]}{P2} \right\rceil &{} \text {if } 0<X{_p}[n-1]\le P2 \\ \left\lceil 2^N \times \frac{X{_p}[n-1]-P2}{2^{N-1}-P2} \right\rceil &{} \text {if } P2<X{_p}[n-1]\le 2^{N-1} \\ \left\lceil 2^N \times \frac{2^N - P2 - X{_p}[n-1]}{2^{N-1} - P2} \right\rceil \ &{} \text {if } 2^{N-1}<X{_p}[n-1]\le 2^N - P2 \\ \left\lceil 2^N \times \frac{2^N - X{_p}[n-1]}{P2} \right\rceil &{} \text {if } 2^N - P2<X{_p}[n-1]\le 2^N -1 \\ 2^N-1-P2 &{} otherwise\end{array}\right. }. \end{aligned}$$

The values created by the recursive cells in the internal state *Xs*[*n*], *Xp*[*n*]are sent to the output function. The output sequence *Xg*(*n*) is then created via chaotic multiplexing controlled by the chaotic sequence $Xth = X1\_s(n-1)\oplus X1\_p(n-1)$ and by a threshold $Th=2^{N-1}$, as illustrated in Eq. (3), or by xoring $X1\_{s}$ and $X1\_{p}$ as clarified in Eq. (4).

3 $$\begin{aligned}&Xg(n)=\left\{ \begin{array}{cl} X\_s(n),&{} \hbox { if}\ 0 < Xth \le Th\\ X\_p(n),&{} \text{ otherwise } \end{array}\right. \end{aligned}$$

4 $$\begin{aligned}&Xg(n)=X_s(n)\oplus X_p(n). \end{aligned}$$

The national institute of standards and technology (NIST) statistical test^27^, among the most widely used tests for analyzing the randomization of binary streams, is used to examine the statistical correctness of the keystream created. This is a statistics suite comprising 188 tests and subtests for evaluating the randomization of random binary sequences. These tests look for several sorts of non-randomness in the sequence. Within each secret key, 100 different binary sequences have been created and 31,250 samples (equal to one million bits) were then evaluated using the NIST tests. For each test, a set of 100 $P\ value$ is generated, and a sequence passes a test when the $P\_value\ge \alpha =0.01$, where $\alpha$ is the test’s threshold of significance. The setting of $\alpha = 0.01$ indicates that 1 percent of the 100 produced sequences are likely to fail. The percentage of produced sequences that pass a test is equal to the number of $P\_value\ge \alpha$ divided by 100. The acquired proportion versus test for delay 1 is displayed in Fig. 8. All of the tests and sub-tests pass the national institute of standards and technology (NIST) tests, as demonstrated.With the exception of the random excursions variation test, the minimum pass rate for each statistical test for 100 binary data is about equal to 0.960150. The random excursions variance testing has a pass rate of around 0.952091 for a sample size of 62 binary data.

### Measuring lightweight characteristics in software

#### Power and energy consumption

Reduced power consumption is a critical component of lightweight systems. Power consumption is crucial since the gadgets we’re targeting have a short battery life, thus consuming power reduces their lifespan. We must measure the power to see whether it is suitable for Lightweight systems. We use the following tools to measure energy and power usage.

- RAPL The Intel Running Average Power Limit (RAPL) interface is an effective tool for measuring energy and power usage^28^. As part of the power-coping interface, RAPL is implemented in the Linux kernel. It measures the CPU and RAM’s energy consumption precisely. RAPL precisely monitors the energy spent by a physical component, followed by the energy consumed by the code executing on that component and the processes .
- PowerTop tool PowerTop is a Linux utility offered by Intel. PowerTOP is a tool for measuring and explaining power use. It is also used to reduce power usage by experimenting with different power management settings^29^. These power management settings indicate how well the various hardware power-saving capabilities are employed. We can quickly define a program’s power inefficiencies using PowerTOP.

#### Memory usage

Memory utilization reflects the fact that limited resource devices have a tiny memory size. Due to limited memory use, we must reduce the size of the encryption system. Also, because embedded systems have limited RAM and ROM, a smaller cryptosystem provides us more alternatives for selecting the CPU, lowering the cost. The FELICS framework is used to measure memory utilization.

*FELICS framework* The Fair Evaluation of Lightweight Cryptographic Systems (FELICS) framework is a free and open-source benchmarking framework for Linux operating systems. A benchmarking framework is a framework for comparing standards and producing exact results. FELICS is a lightweight encryption software implementation. This implementation compares the performance of several IoT embedded devices under identical use scenarios. It also compares the new encryption to earlier ciphers using different measurement circumstances. FELICS may be used to evaluate lightweight stream or block ciphers by implementing C code or assembly. Then we may assess three metrics: execution time, code size, and RAM usage. In the evaluation, FELICS may be implemented on three devices: 32-bit ARM, 16-bit MSP, and 8-bit AVR^30^.

#### Enhancements on the current system

**Figure 4:**
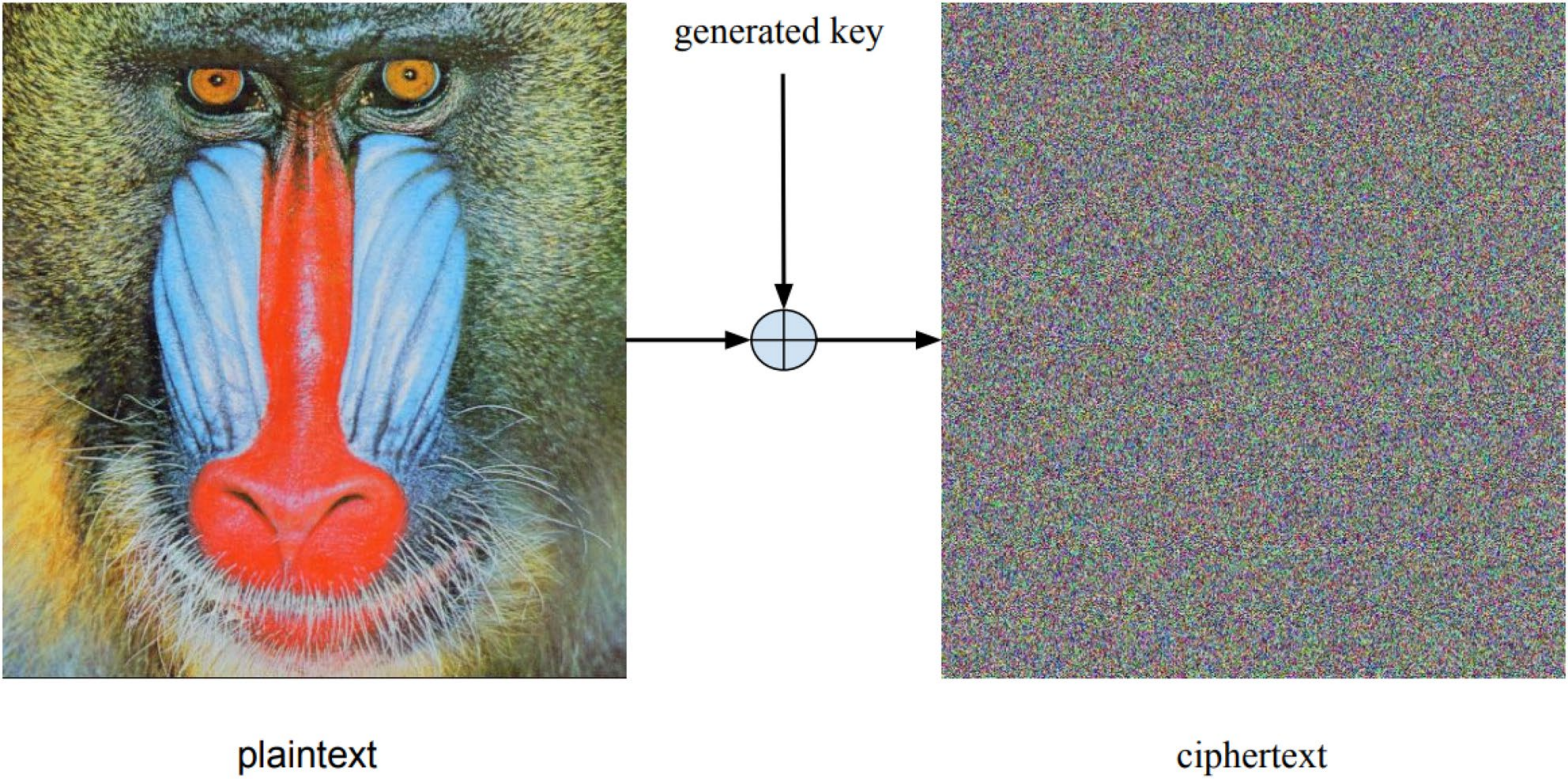
Stream cipher encryption for a plaintext image with it is cipher image^31^.

The current technique encrypts many types of data with a chaos-based stream cipher algorithm. To generate a new key, the system encrypts the plaintext with a secret key and an initial vector (IV). The length of the key stream is more than or equivalent to the length of the plaintext. As seen in Fig. 4^31^, deploying XOR on the plaintext and the produced key provides the ciphertext. Both the transmitter and the recipient must have the same secret key and IV. The system generates the keys through the use of two chaotic maps: the discrete skew tent map and the discrete piecewise linear chaotic map (PWLCM). The internal state of the system contains one, two, and three delays, as well as two non-linear filters. Both recursive filters use linear feedback shift register (LFSR) technology to achieve the necessary confusion.

#### Encryption operation

To test the suggested system in the reality, the hardware implementation is done on FPGA-board using the Verilog language. The hardware devices only have a limited number of resources. As a result, we estimate the suggested hardware system’s resource consumption in order to test its execution capability on these devices. Real-time and hardware applications require strong performance and appropriate throughput to finish their jobs on time, thus we assess their throughput to check the system performance in hardware.

#### Result and analysis comparison

We demonstrate that the improved system satisfies the criteria of lightweight cryptosystems. We also demonstrate how the technology may be utilized in FPGA boards and IoT systems. The suggested system’s result was reached by using the previously described tools as well as hardware implementation using the FPGA-board and Verilog language. These findings are compared to those of other LWC and hardware implementations.

## Software and hardware implementation

### Software implementation

In this part, we will go over the software implementation. It is accomplished by employing the tools stated before in the Design and methodology section on the system.

#### Power

We utilized the RAPL tool to test power consumption in order to determine whether the method needed little power to encrypt the data. Using the tool’s data, we were able to determine whether the power usage is appropriate. Furthermore, we compared the findings to various stream cipher methods’ power usage.

Using the RAPL interface, we tested the power consumption of our proposed Chaos-Based Stream Cipher algorithm^32^. The average energy usage findings of the proposed system were then compared to four different stream cipher techniques. Those stream cryptosysem are CM-SC^33^, HC-128, CS-SC^34^ and Rabbit. The power consumption comparison is shown in Table 1.

**Table 1:** RAPL power consumption (J).

Stream cipher	Proposed (our proposed system)	CM-SC	CS-SC	Rabbit	HC-128
PKG energy (J)^32^	0.035995	0.078613	0.022672	0.013855	0.038768
PP0 (J)	0.005281	0.036316	0.010297	0.006104	0.020316
PP1 (J)	0.003505	0.007568	0.000112	0.000150	0.000030
DRAM (J)	0.002807	0.012939	0.002669	0.001648	0.003806

Each RAPL result creates four values. The first is the PKG, which receives the total power usage (CPU) in Joules. Following that is the PP0, which indicates the PowerPlane0 (core) for the power consumption of the CPU cores only. PP1 on the other hand, stands to uncore power usage. Finally, the DRAM measurements show the amount of power consumed by the memory controller^35^.

#### Memory (RAM and ROM)

We chose the FELICS tool to estimate memory usage since it is a benchmarking framework that provides us with an accurate memory footprint. The amount of memory that a software can utilize while executing is referred to as its memory footprint. We assessed the memory usage to see if it was low enough to be considered a lightweight cryptosystem and then compared it to other ciphering systems.

We had to download the prerequisites that the FELICS framework relies on in order to obtain it. The requirements are the AVR microcontroller, the MSP debugger, and the ARM drivers, which we obtained by running the following commands:

- sudo apt-get install gcc-Avr
- sudo apt-get install gcc-msp430
- sudo apt-get install gcc-arm-none-eabi.

Following that, we completed the necessary settings to set up the framework’s core module. We had the framework ready at this point, but in order to evaluate memory usage, we have to follow the framework guidelines. The FELICS framework mandates that the algorithm and its files have a certain structure. These files must perform certain functions in a specific order, as indicated by the templates provided by the framework. Using this framework, we may create the cipher in a variety of settings and test cases.

The architecture separates the algorithm primarily into two parts: encryption and setup. We might include more files for our algorithm or code in each section. The setup section provides the code required to obtain the key, IV, and settings required for the encryption procedure. Simultaneously, the encryption section includes the code that creates the key and performs the encryption operation. We must be careful where and how we implement the code in the framework since each modification impacts the RAM or ROM utilization.

FELICS displays the ROM result as a code size in a table that also displays the code size of each file utilized in the algorithm. The code size quantifies the amount of data stored in the target device’s Flash memory^36^. To calculate the overall ROM usage, we add the data from the table. The RAM consumption, on the other hand, covers both the stack needs and the data requirement result. This result is presented in the form of a table, which indicates the RAM sizes of the key, IV, state, data, encryption, and setup. To calculate the overall RAM utilization, we add the values from the table.

### Hardware implementation

On this part, we show how to implement the suggested method in FPGA using the Verilog programming language. We also demonstrate the algorithm’s performance evaluation.

#### Utilization

On the FPGA board, we assess the suggested algorithm’s resource use (Spartan- xc6slx75-2-csg484). To that aim, we implement the suggested method in Verilog so that it may be used in real-world hardware applications.

The Algorithm is built in VHDL using Xilinx ISE Design Suite 14.6^37^, and the VHDL code is then synthesised using the Xilinx ISE Design Suite. Following the synthesis procedure, the Xilinx ISE Design Suite provides reports. The reports describe resource usage, time, and so forth. We are considered in the resource usage report.

#### Performance

We need to assess the throughput and the clock in order to evaluate the performance of the proposed system. The throughput of a system is the number of units of information it can handle in a given length of time. In our situation, this is how many bits per second. Using the findings of the FPGA board (Spartan- xc6slx75-2-csg484), we were able to determine whether or not the performance is satisfactory.

## Results

We examine the results and compare them to other systems; we also determine whether the system meets our objectives by comparing it to lightweight criteria.

### Software result

#### Power

The average energy value is computed after executing the proposed technique 100 times on the standard Lena picture (256 256 3) with 100 distinct secret keys.

**Figure 5:**
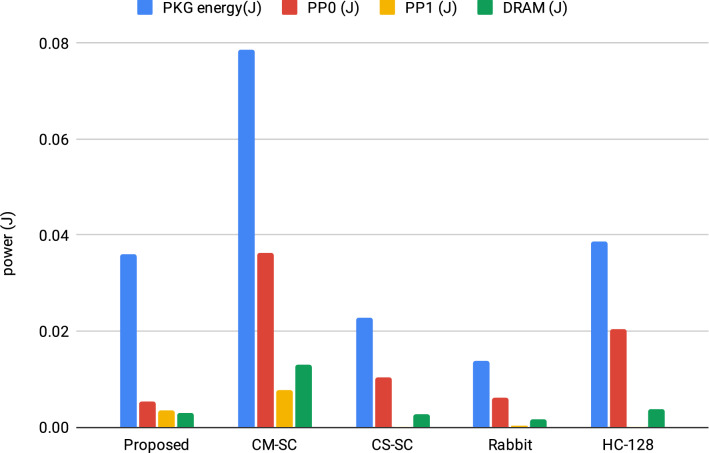
The comparison of the RAPL power consumption results.

We make a comparative study between acquired power consumption findings with latest stream crypto-system in Table 1 (for image size equals 256 256 3). In Fig. 5, we can observe that:

- Our suggested system consumes less power overall (PKG energy) than CM-SC and HC-128.
- Our proposed system uses the least power in the core power consumption (PP0).
- Our suggested system uses less power than CM-SC in the uncore power consumption (PP1).
- Our suggested DRAM system uses less power than CM-SC and HC-128.

The preceding findings demonstrate that our system consumes less power than other stream ciphers.

#### Memory (RAM and ROM)

Using the specified test vectors, the first scenario (Scenario 0) is examined. The second scenario (Scenario 1) involves encrypting 128 bytes of data. It addresses the demand for secure communication between sensor networks and IoT devices. The second scenario is used to generate the results (Scenario 1).

**Figure 6:**
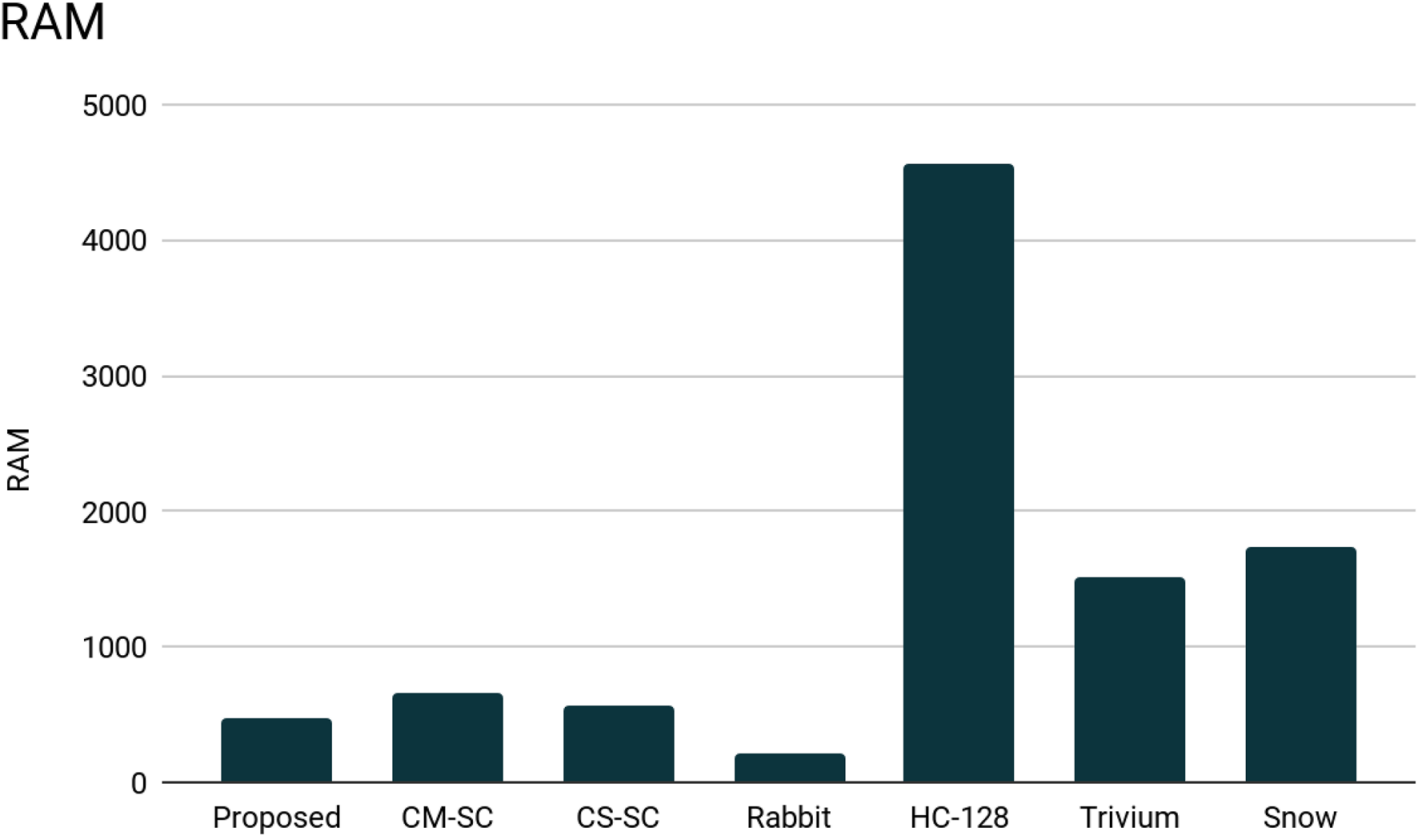
The comparison of the RAM consumption of the proposed system with different encryption algorithms.

**Figure 7:**
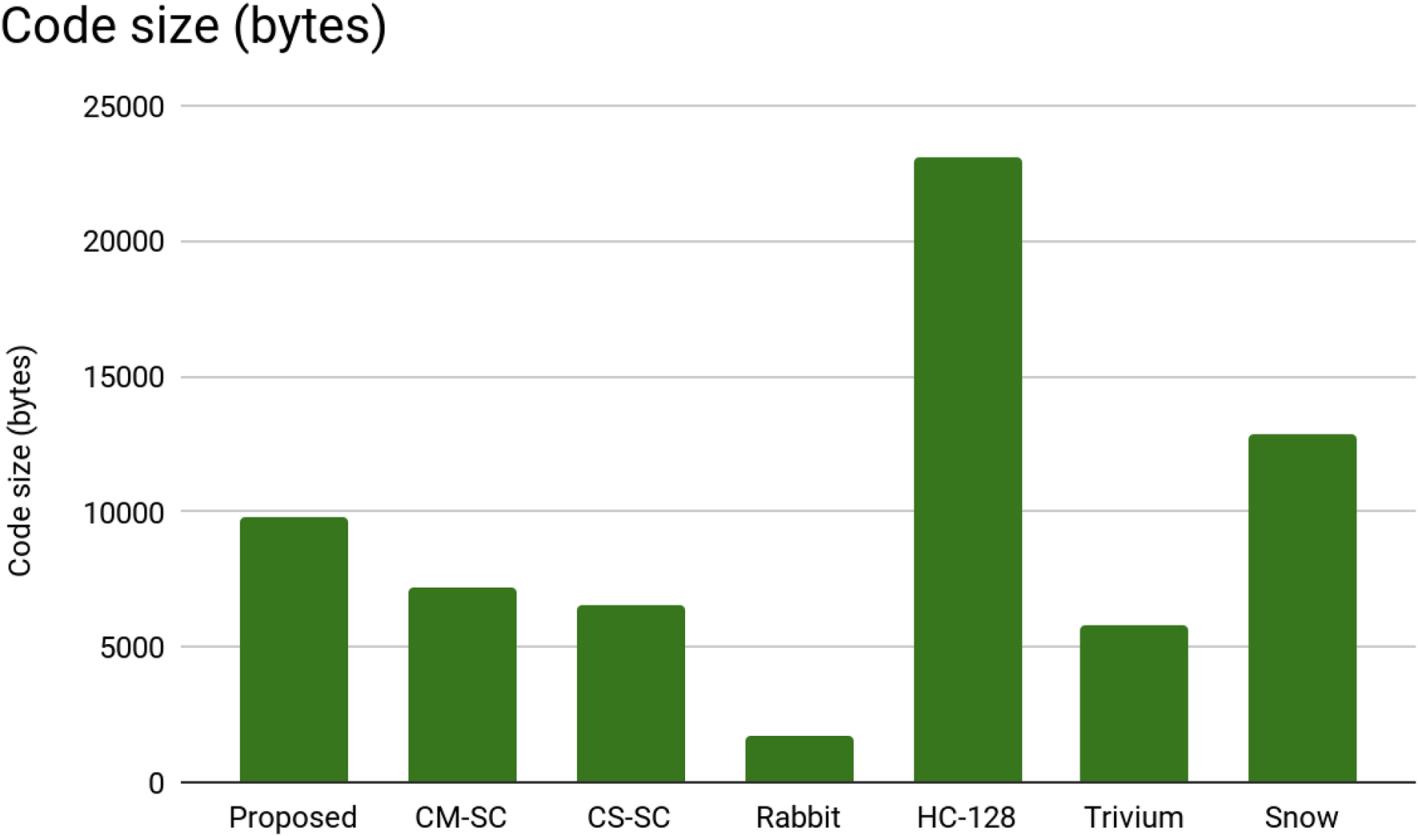
The comparison of the code size (ROM) in the proposed system with different encryption algorithms consumption.

In Table 2, we compare our obtained RAM and ROM (memory) consumption results with some of the known stream ciphers. As we can see in Figs. 6 and 7:

- In the code size (ROM), our stream cipher consumes fewer bytes than HC-128 and Snow stream ciphers.
- In the RAM consumption, our algorithm consumes less memory than 5 of the other stream ciphers. It’s less than CM-SC, CS-SC, HC-128, Trivium, and Snow stream ciphers.

The experimental findings suggest that our system consumes less memory than other stream ciphers. They also show that the suggested method consumes less than 8 KB of RAM and 32 KB of ROM. This indicates that the suggested approach is appropriate for devices with low memory and tiny devices. It is also compatible with IoT devices, which are thought to have limited memory.

**Table 2:** FELICS memory consumption.

Cryptosystem	Size of code (bytes)	Memory
Proposed	9817	468
CM-SC	7240	660
CS-SC	6562	564
Rabbit	1714	216
HC-128	23,100	4556
Trivium	5764	1516
Snow	12,861	1741

### Hardware result

This subsection discusses the results and compares them with the other stream hardware implementations.

#### Utilization

At the end of the experiment, we collect the suggested system’s resource use and compare it to another pseudo chaotic number generator, as shown in Table 3.

**Table 3:** Resource consumption rate of the proposed pseudo chaotic number generator for the FPGA board (Spartan- xc6slx75-2-csg484).

Logic utilization	Available	Proposed		^38^	
		Used	Utilization (%)	Used	Utilization (%)
Number of slice Registers	93,296	38	0.04	278	0.30
Number of slice LUTs	46,648	26,631	57.09	19,283	41.34
Number of fully used LUT-FF pairs	19,361	2	0.01	200	1.03
Number of bonded IOBs	328	65	19.82	36	10.98
Number of BUFG/BUFGMUXs	16	1	6.25	1	6.25
Number of DSP48A1s	132	39	29.55	25	18.94

Table 3 shows the consumption of hardware resources by the proposed system and another system^38^. The results denote that the proposed system consumes less resources. They also show that the proposed system consumes less slice registers and the fully used LUT-FF pairs than the other system. But the proposed system consumes more Number of Slice LUTs and more number of bonded IOBs than the other system.

#### Performance

Because the time required to determine the clock frequency is included in the timing report, we can calculate the throughput using the clock frequency by using the equation below. The suggested system has a throughput of 178.304 Mbps for N = 32 and a clock frequency of 5.572 (in Xilinx Spartan 6 platform), as shown in Table 4.

5 $$\begin{aligned} Throughput(Mbps)= N \times Clock frequency. \end{aligned}$$

**Table 4:** Performance comparison between the proposed system to other systems.

Paper	Platform	Clock (MHz)	Resources	Output bits)	Mbps
Ours	Xilinx Kintex 7	1178.967	2 FFs, 26,612 LUTs	32	37,726.944
	Xilinx Virtex 6	1204.529	2 FFs, 26,612 LUTs	32	38,544.928
	Xilinx Spartan 6	599.323	2 FFs, 26,631 LUTs	32	19,178.336
^38^	Xilinx Kintex 7	12.562	199 FFs, 19,260 LUTs	32	401.984
	Xilinx Virtex 6	11.706	19,266 LUTs	32	374.592
	Xilinx Spartan 6	5.527	200 FFs, 19,283 LUTs	32	176.864
^39^	Xilinx Kintex 7	59.492	133 FFs, 311 LUTs	–	125 (kbps)
^39^	Xilinx Virtex 6	90.2445628	43,732 LUTs	32	58.7

As shown in Table 4, we can get the throughput of the proposed system in different FPGA boards and compare them with other systems. According to the table, the proposed system has a higher throughput than the other mentioned systems. In addition to the good throughput, the proposed system occupies a small implementation area, which reduces the hardware resource consumption. In conclusion, the proposed system is applicable to real hardware and IoT applications, with high performance (throughput) and low resource consumption.

### Hardware security

Several tests must be performed to determine the convergence of the proposed system with pseudo sequences generator. Every test assesses a unique attribute, such as the relationship between random and produced sequences. The National Institute of Standards and Technology (NIST) tests are also used to determine how near the produced sequence is to a random sequence. Furthermore, we ran many security tests to demonstrate our model’s resistance to attacks^40^.

#### Phase space test

The phase space test is applied to the proposed system in^41^ , as well as the mapping of a sequence X(n) formed by 3,125,000 samples out of the 3,125,100 samples generated to reach the transitional regime Tr = 100, as well as the mapping of 1000 samples from the random X(n). The results of the tests demonstrate that the region appears to be utterly disordered, with no relationship between surrounding sample values.

#### Histogram and chi-square tests

**Figure 8:**
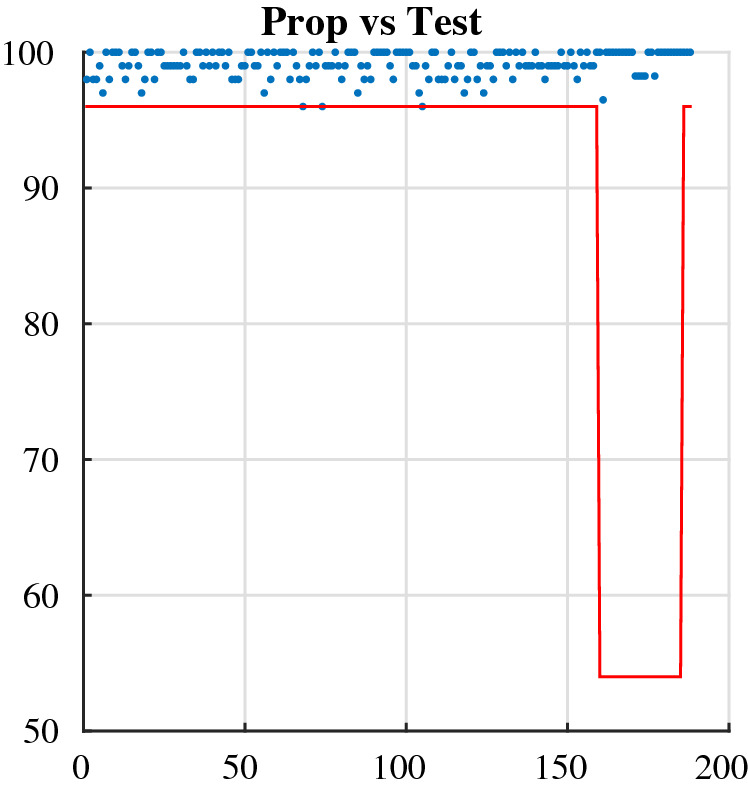
NIST of the acquired key-stream sequences^41^.

The pseudo number generator should produce sequences with a uniform distribution. The histogram of the created sequence X(n) in^41^, which must be checked by the stated chi-square test. The experimental value of the chi-square test is smaller than the theoretical value, confirming the uniformity of the histogram (Fig. 8).

#### NIST test

The generated sequences from the pseudo number generator have to pass the statistical NIST test that contain 188 test ans sub-test that evaluate the randomness of long binary sequence. The proposed system tested on the NIST test in^7,41^ and their is a result for 15 statistical tests. According to the final results the proposed system generated unrecognizable sequences from random sequences.

#### Keyspace

The bigger keyspace method, as we know in encryption, has a high capacity to withstand a brute force attack, whereas algorithms with a limited key space suffer from a lack of sequence randomization. The suggested cryptosystem has distinct keyspaces depending on the delay. The keyspace is about 299 bits in delay = 1, but it grows to 555 bits in delay = 3. Because of the large number of keyspaces, the proposed cryptosystem is resistant to brute force attacks.

#### Key security and sensitivity attack

We used two important matrices to evaluate the key sensitivity of our proposed cryptosystem: the amount of pixel change rate (NPCR) and the unified Average changing intensity (UACI), which show that the proposed proposal is very sensitive to a one-bit change that appears when we encrypt the “Lena” image more than 100 times using 100 secret keys with a difference in the least significant bit (LSB).

6 $$\begin{aligned} NPCR = \frac{1}{L \times C \times P}\sum \limits _{p=1}^P\sum \limits _{i=1}^L \sum \limits _{j=1}^C D(i,j,p)\times 100\% \end{aligned}$$

Where

7 $$\begin{aligned} D(i,j,p) = \bigg \{ \begin{array}{l} 0 , if C_1 (i,j,p)=C_2 (i,j,p) \\ 1 , if C_1 (i,j,p) \ne C_2 (i,j,p) \\ \end{array} \end{aligned}$$

And UACI, which is used to calculate the average intensity difference between two ciphered images, is defined as follows:

8 $$\begin{aligned} UACI = \frac{1}{L \times C \times P\times 255} \sum \limits _{p=1}^P\sum \limits _{i=1}^L \sum \limits _{j=1}^C \mid C_1(i,j,p)-C_2(i,j,p)\mid \times 100\% \end{aligned}$$

In the preceding equations, L, C, and P represent the image’s length, width, and plane size, respectively. I, j, and p represent the row, column, and plane indexes, respectively. As demonstrated in Table 5, the suggested cryptosystem’s NPCR and UACI values are close to the ideal NPCR and UACI values of 99.61 and 33.46, respectively^42^.

**Table 5:** The results of NPCR and UACI matrices.

Algorithm	NPCR	UACI
Proposed system	99.655	33.439

#### Information entropy

In information theory, the entropy E(X) is a statistical measure of uncertainty^43^. This is how it is defined:

9 $$\begin{aligned} E = - \sum \limits _{i=0}^{255}P(x_{i})\log _{2}P(x_{i}) \end{aligned}$$

Where X is a random variable and p(xi) is the probability of the gray value xi occuring. Assume there are 256 states of the information source with the same chance in the red, green, and blue colours of the picture. We can obtain an ideal E(X) = 8, which is a completely random source. As shown from Table 6 the information entropy of various Cipher Images such as Titanic, Photographer, Manhattan, Camera-man, Lena, and Boat is closer to 8. Entropy values approaching 8 are found in all ciphers, suggesting that the cipher image is a random collection of pixels.

**Table 6:** Information entropy of some cipher images.

Ciphered image	Titanic	Sharukhan^41^	Manhattan	Photographer^41^	Camera-man	Lena	Boat^41^
Results of Entropy	7.999	7.999	7.999	7.999	7.999	7.999	7.999

#### Correlation analysis

The correlation evaluation is very crucial, especially in the encryption field, because it is important to hide information from an attacker who may know some information of the plaintext from the encrypted data, so in order to guarantee security, the correlation of the presented stream cipher must be as low as possible. We run this test by feeding 10000 adjacent pixels from the plane picture and the encrypted image as input to the Eqs. (10), (11), (12) and (13).

10 $$\begin{aligned} P_{xy} & = \frac{Cov(x,y) }{\sqrt{D(x)}\sqrt{D(y)}} \end{aligned}$$

11 $$\begin{aligned} Cov(x,y) & = \frac{1 }{N} \sum \limits _{i=1}^{N}([x_{i}-E(x)][y_{i}-E(y)] ) \end{aligned}$$

12 $$\begin{aligned} D(x) & = \frac{1 }{N} \sum \limits _{i=1}^{N}(x_{i}-E(x))^{2} \end{aligned}$$

13 $$\begin{aligned} E(x) & = \frac{1 }{N} \sum \limits _{i=1}^{N}(x_{i}) \end{aligned}$$

**Figure 9:**
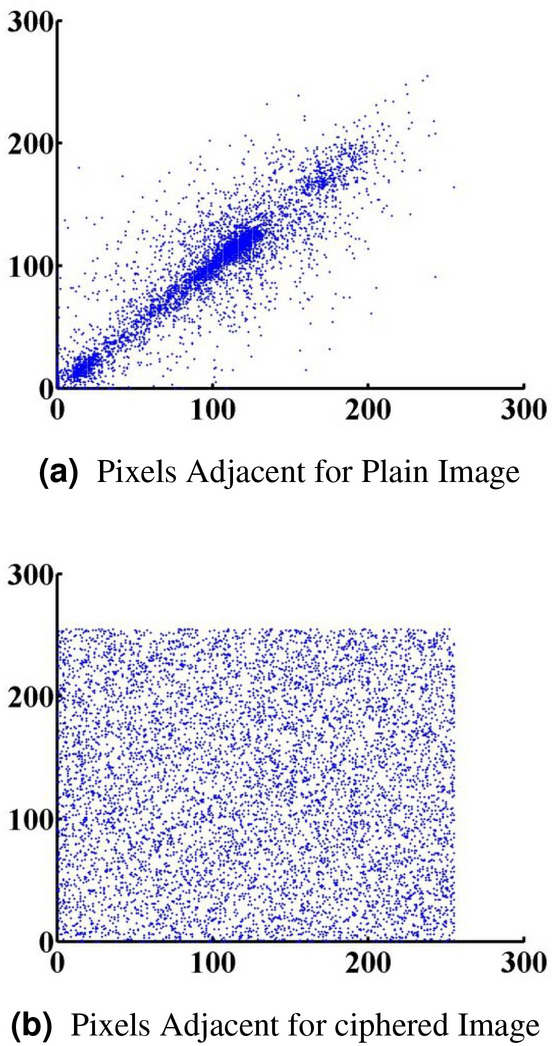
Pixels adjacent for plain and cipher image^41^.

In the previous equations, *pxy* represents the correlation coefficient of two sequences, x and y. The x and y values are Xi and Yi, respectively. To demonstrate that the cipher picture is not the same as the original image, we utilized correlation analysis of surrounding pixels for both the cipher and the original image. Figure 9a demonstrates that adjoining pixels in the plain picture are redundant and correlated, but adjacent pixels in the cipher image appear to have as little redundancy and correlation as possible. This is simply one more illustration of how our proposed strategy is resistant to statistical assaults.

## Conclusion and future work

### Conclusion

We developed a secure lightweight cryptosystem for IoT and tiny devices in this study. The technology consumes less power, extending the life of these gadgets. This system also requires less memory, making it ideal for IoT and tiny devices (often with less than 8k RAM (Random Access Memory) and 32k ROM) (Read Only Memory). The study showed a viable hardware lightweight cryptosystem built on an FPGA board using the Verilog programming language. The suggested hardware solution is based on resource usage that is appropriate for real-time applications and IoT devices. This system also shown good performance with large throughputs to enable real-time data delivery.

### Future work

In the future, the system may be modified to focus on the area of interest (ROI), making the encryption process even faster than previously. Furthermore, implementing an encrypted mobile application will be a useful step because the system is currently lightweight and works well on mobile devices. In addition, we propose a lightweight system using a one-way hashing function.
